# Discriminating between natural and anthropogenic earthquakes: insights from the Emilia Romagna (Italy) 2012 seismic sequence

**DOI:** 10.1038/s41598-017-00379-2

**Published:** 2017-03-21

**Authors:** Matteo Albano, Salvatore Barba, Gabriele Tarabusi, Michele Saroli, Salvatore Stramondo

**Affiliations:** 1 Istituto Nazionale di Geofisica e Vulcanologia, Via di Vigna Murata 605, 00143 Roma, Italy; 2 Dipartimento di Ingegneria Civile e Meccanica, Università degli Studi di Cassino e del Lazio Meridionale, Via G. di Biasio 43, 03043 Cassino, Italy

## Abstract

The potential for oilfield activities to trigger earthquakes in seismogenic areas has been hotly debated. Our model compares the stress changes from remote water injection and a natural earthquake, both of which occurred in northern Italy in recent years, and their potential effects on a nearby Mw 5.9 earthquake that occurred in 2012. First, we calculate the Coulomb stress from 20 years of fluid injection in a nearby oilfield by using a poroelastic model. Then, we compute the stress changes for a 2011 Mw 4.5 earthquake that occurred close to the area of the 2012 mainshock. We found that anthropogenic activities produced an effect that was less than 10% of that generated by the Mw 4.5 earthquake. Therefore, the 2012 earthquake was likely associated with a natural stress increase. The probability of triggering depends on the magnitude of recent earthquakes, the amount of injected water, the distance from an event, and the proximity to the failure of the activated fault. Determining changes that are associated with seismic hazards requires poroelastic area-specific models that include both tectonic and anthropogenic activities. This comprehensive approach is particularly important when assessing the risk of triggered seismicity near densely populated areas.

## Introduction

In recent decades, increasing numbers of anthropogenic earthquakes have caused unexpected damages^1, 2^, worrying populations around the world and requiring better management of the increased risk. Several scientific studies have modelled the physical phenomena that are associated with increased anthropogenic earthquakes and seismic hazards^1, 3–9^. However, different approaches have led to different results and interpretations, suggesting that the descriptions of physical phenomena are subject to considerable uncertainty. Moreover, assessments of the relative seismic risk that is associated with natural and anthropogenic causes are often accompanied by scepticism in the scientific community^10^. We investigate the relationship between hydrocarbon activities in northern Italy and the initiation of a nearby Mw 5.9 earthquake that occurred in 2012. Given the lack of knowledge regarding the crustal stress levels and rupture thresholds, we compare the stress perturbations from the hydrocarbon activities with those from an earlier natural earthquake of Mw 4.5 that occurred in 2011. This comparison enables us to analyse whether the triggering mechanism of the 2012 earthquake was natural or anthropogenic.

Anthropogenic earthquakes have been widely acknowledged by the scientific community^11^ and are commonly referred to as induced or triggered seismicity^12^. These earthquakes are labelled induced when anthropogenic activities significantly perturb *in situ* stresses in small rock volumes far from large pre-existing faults. Both the number and magnitude of earthquakes increase with the total injected or extracted fluid volume^4, 13^. Earthquakes are labelled as triggered when industrial activities perturb tectonic structures that are already affected by crustal stresses. In this case, small stress changes can be significant because they disturb unstable (nearly critical) fault systems and may stimulate large earthquakes. The maximum size of a triggered earthquake depends on the total volume of injected fluid and the size of the affected tectonic structures^14^.

The increases in earthquake rates and seismic hazards in recent decades have been associated with the growth of certain industrial activities^12, 15, 16^. Such activities include mining^17^, hydrocarbon production, fluid withdrawal or injection^18^, drilling, hydrofracturing^19^, geothermal operations^20^, and reservoir impoundment^21^. Assessing these new and frequent seismic hazards requires a better understanding of the associated earthquake generation mechanisms in both known seismic areas and areas that are commonly considered aseismic^13^.

The occurrence of induced or triggered seismicity has often been associated with or attributed to the production of hydrocarbons^15, 18, 22^. In the hydrocarbon industry, hydraulic fracturing and crude oil extraction are acknowledged as common causes of induced earthquakes; however, wastewater disposal in deep formations can weaken pre-existing faults and trigger large earthquakes^2, 13^.

The presence of active faults makes anthropogenic earthquakes difficult to study. In fact, the generation mechanisms and magnitudes of triggered earthquakes depend on several factors. These factors include the volume of the injected fluids, the extent of the perturbation^13^, the geometries and orientations of the fault planes, the hydraulic connections between the injection and extraction zones and the fault planes^15^, and the magnitude of the tectonic stress field.

Fault rupturing and the likelihood of subsequent seismicity are often modelled by using the Mohr-Coulomb failure criterion^23^. In this case, the critical shear stress τ_c_ (i.e., the stress that causes the fault to slip) is proportional to the normal stress σ according to equation (1):

1 $${\tau }_{c}=c+\mu \cdot \sigma $$

where c is the inherent shear strength of the rock, μ is the coefficient of internal friction, and σ is the normal stress. According to this criterion, fault failure occurs when the shear stress τ reaches the critical value τ_c_, i.e., when the normal stress decreases or the shear stress increases^12^.

The sum of the changes in the normal and shear stresses defines the Coulomb stress change (hereinafter ΔCFS; see the Methods section for details). The spatial distribution of early aftershocks is approximately correlated with the coseismic Coulomb stress change of major natural earthquakes. Aftershocks are more likely to occur where the static stress increases and less likely where the stress decreases^23–25^. This behaviour suggests that earthquakes advance toward failure in regions of increased static stress.

Fault parameterization controls the ΔCFS; however, the input data, model assumptions, and modelling procedures that are used in source models have inherent uncertainties. These uncertainties propagate into random and systematic uncertainties in the calculated stress changes. Additionally, these uncertainties are often underestimated or neglected, rendering the inferred stress changes unreliable^24^.

The Coulomb stress approach has also been used to study anthropogenic earthquakes^26, 27^. In these cases, point-like fluid injection sources are used, often with a detailed knowledge of subsurface properties, at least near the source. The modelled stress perturbations are typically smaller than natural crustal stresses, except in some cases near the injection point. Anthropogenic operations such as fluid injection/extraction are time-dependent processes because of operational constraints; however, the ΔCFS also depends on the subsequent crustal pore fluid diffusion^28, 29^ far from the injection point. Accordingly, appropriate hydraulic and strength parameters must be selected for the model.

Additionally, the fault stress before the perturbation is unknown and difficult to determine. The *in situ* fault stress is particularly difficult to define; thus, determining whether a particular anthropogenic disturbance in the Coulomb stress is of practical use is challenging. This uncertainty may also create difficulty in determining whether an earthquake results from natural or anthropogenic causes, especially in seismically active areas. ΔCFS values as low as 0.01 MPa can trigger earthquakes^23, 30^, as can higher ΔCFS values^31^. Such small values suggest that the faults that were involved in the event were nearly critically stressed previously^7^.

The 2012 earthquake sequence that occurred in the Emilia-Romagna region (Italy) is representative of such an event. This event had relevant social, cultural, emotional and economic effects and caused severe damage in many localities, especially to historical centres and factories, resulting in more than 4 billion euros of damage^32^. Additionally, twenty-seven people were killed, hundreds of people were injured, and more than 40,000 were evacuated.

**Figure 1:**
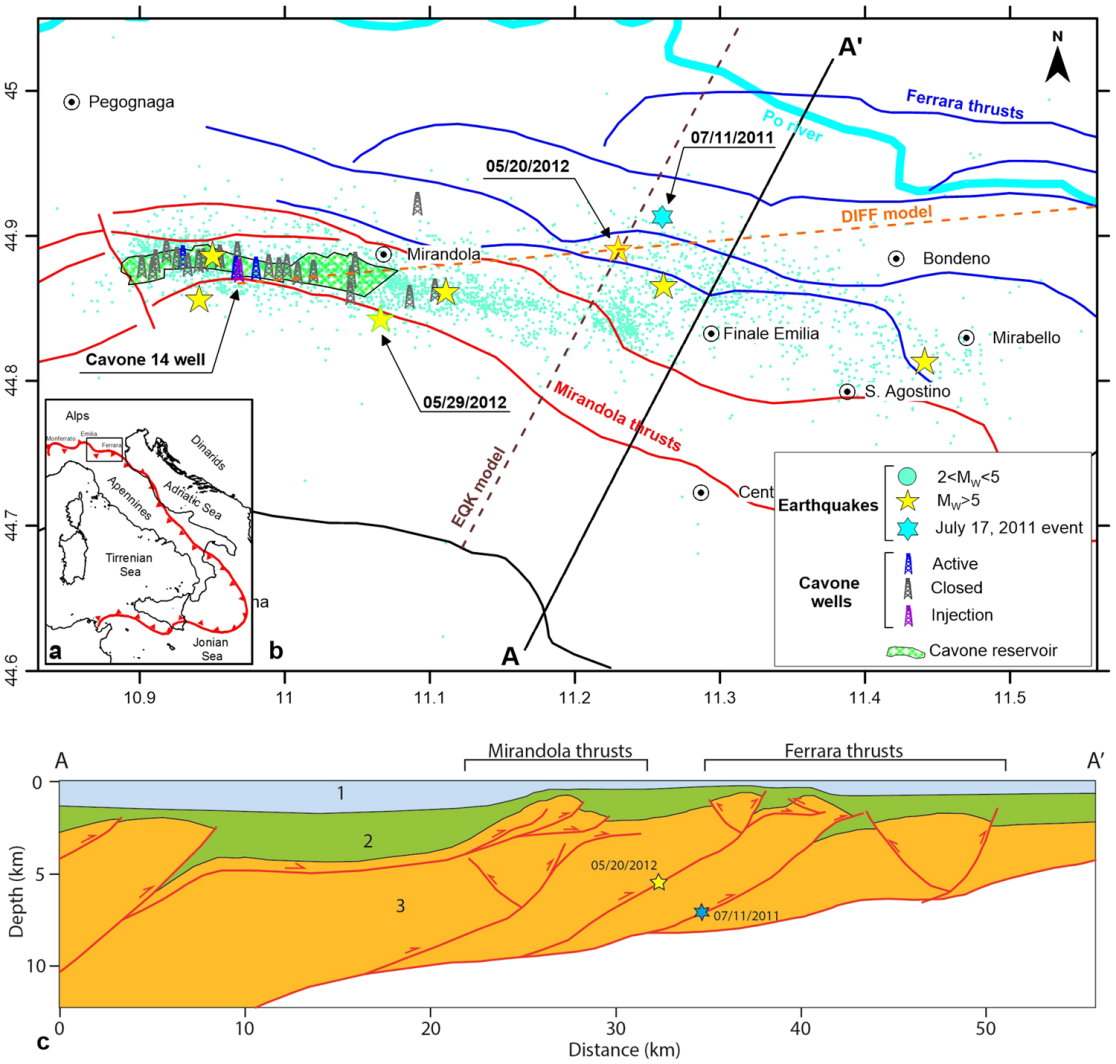
(**a**) Main thrust alignment in Italy. (**b**) Detailed map of the study area (black box in panel a). Location of the 2012 Emilia seismic sequence with respect to the Cavone oilfield and the production/injection wells. The yellow stars are earthquakes larger than M_L_ > 5.0. The two mainshocks are represented by yellow stars with red (05/20/2012 event) and green (05/29/2012 event) outlines. The cyan star indicates the position of the July 17, 2011 event. The tectonic structures represent the main thrusts (continuous lines) and back-thrusts (dashed lines) of the Ferrara (blue), Mirandola (red) and Pede-Apennines (black) systems^75^. The orange and brown dashed lines represent the cross sections of the DIFF and EQK models, respectively. (**c**) Simplified geological cross-section of A-A’ in panel b. The units in this section are as follows: 1- Quaternary layer, 2- Pliocenic-Miocenic layer, and 3- Triassic-Lower Cretaceous layer. This figure was created with Surfer® from Golden Software, LLC (www.goldensoftware.com) and Adobe Illustrator (www.adobe.com).

The Emilia-Romagna region is characterized by an NE-verging thrust system^33, 34^ (Fig. 1a) that originated from the Cenozoic collision between the European plate and the Adria plate^35^. These thrusts represent the frontal portion of the Northern Apennines fold-and-thrust belt^36^ and are assumed to generate earthquakes up to magnitude 6.1 at depths of 2–10 km^37, 38^. The largest historical earthquakes occurred more than 500 years ago (e.g., Mw 5.5 on November 17, 1570 and Mw 4.7 on March 17, 1574)^39^.

The Emilia seismic sequence has been attributed to the activation of two segments of the Ferrara-Romagna thrust system: the Ferrara thrust, which was responsible for the Mw 5.9 event on May 20, 2012, and the Mirandola thrust, which was responsible for the Mw 5.7 event on May 29, 2012 (Fig. 1b and c)^40, 41^.

These deadly earthquakes have drawn considerable attention from around the world because of the potential connection between the mainshock rupture and the hydrocarbon activities on the Po plain^42^. In fact, the seismic sequence occurred along an active thrust system that partially overlaps the Cavone oilfield (Fig. 1b), where hydrocarbon-related activities have occurred since 1980. The oil production in Cavone peaked in 1982 and then declined because of the depletion of the reservoir (Supplementary Figure S1). Twenty-two wells have been drilled, and the most recent data (2014) reported that four wells are still productive^43^. Since 1992, an increasing amount of water has been extracted with crude oil and reinjected via the Cavone 14 well (the purple well in Fig. 1b). The injection depth is deeper than the production well depth, terminating in the deep aquifer beneath the oil/water interface in the crystalline basement at a depth of approximately 3335 m^43^. Wastewater reinjection activities continued until before the mainshock, with a mean yearly injection rate of approximately 1.3 × 10^5^ m^3^/yr and a peak of 2 × 10^5^ m^3^/yr in 2004 ^44^ (Supplementary Figure S1).

The effects of the depletion and injection activities at the Cavone oilfield have been studied by using seismological^44^, numerical^45, 46^, and probabilistic approaches^3^. The ICHESE panel^44^ found a correlation between the increased production/injection activities and the seismicity rates before the May 20^th^ event. These researchers concluded that these activities could not be excluded as a potential trigger of the Emilia seismic activity. Further analytical and numerical models^45, 46^ found negligible or negative stress changes in the hypocentre area of the May 20^th^ event. These results exclude static stress changes from injection and production activities as potential triggers of the 2012 earthquake sequence. Similarly, a more recent study^3^ concluded that the depletion-induced stress rate of the Cavone oil field likely did not cause the May 20, 2012 mainshock, and water reinjection was ruled out as a possible triggering mechanism because of the presence of highly impermeable layers.

We focused on water reinjection when studying the Emilia case to investigate the effects of stress changes on nearby tectonic structures from only fluid injection. We used a first-order finite element numerical model to assess the relative effects of anthropogenic activities and natural earthquakes on the stress field at the location of the 2012 Emilia earthquake.

We assumed that the injection well and mainshock causative fault were hydraulically connected, and we neglected the presence of production wells. This choice represents the worst-case scenario. We compared the stress field perturbations with those from natural earthquakes to quantify the effect of injection-induced stress changes. Additionally, we considered the Mw 4.5 event that occurred on July 17, 2011. A search of historical data^47^ showed that this event was the largest reported earthquake in the past 450 years within a radius of 40 km from the 2012 mainshock, thereby constituting our reference earthquake. Additionally, this event activated the same structures that were responsible for the earthquake on May 20, 2012 (see the Methods section for details).

In this comparison, the lack of knowledge regarding the initial stress state is a more relaxed condition because we assume similar geometries and initial stress states for both modelled phenomena. The relative stress changes do not refer to the original state but to the difference between the changes from natural earthquakes and anthropogenic activities. The phenomenon that causes the highest stress increase is the most likely candidate that triggered an earthquake in the area of the May 20, 2012 hypocentre.

The calibrated finite-element model can easily be improved to include geometrical complexities, material heterogeneities, and the dependencies of material parameters on the stress-strain path, allowing the model to be applied to different scenarios with various levels of complexity.

## Results

### Numerical modelling

**Figure 2:**
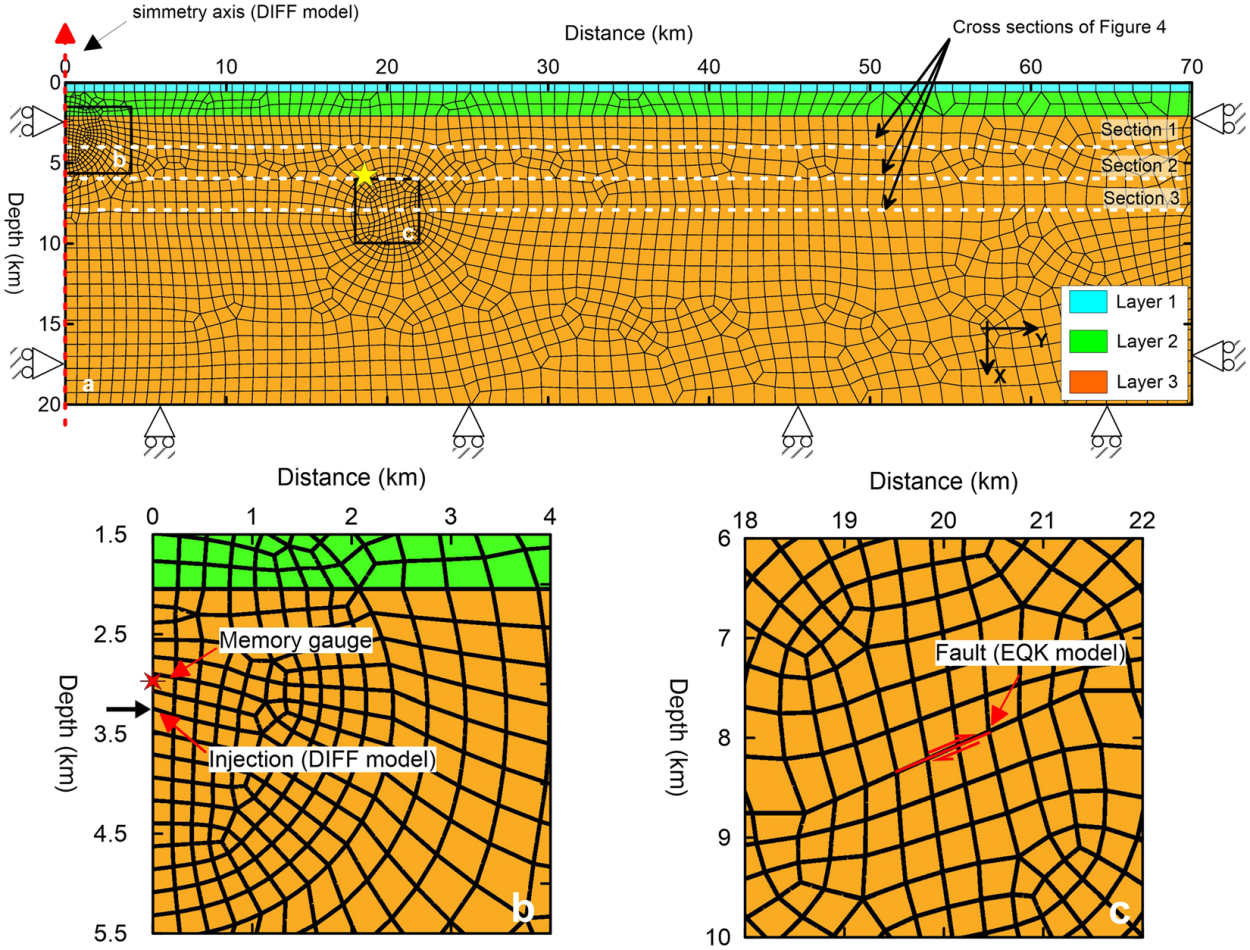
(**a**) Finite element geometry for the DIFF and EQK models. The yellow star identifies the May 20, 2012 earthquake hypocentre. The rollers constrain zero displacement normal to the boundary. The dashed red arrow identifies the symmetry axis of the DIFF model (corresponding to the Cavone 14 well). (**b**) Detailed illustration of the injection area of the DIFF model with the position of the memory gauge^43^. (**c**) Detailed illustration of the fault plane for the simulation of the Mw 4.5 earthquake. These figures were created with Surfer® from Golden Software, LLC (www.goldensoftware.com).

We built a fully coupled, 2D poroelastic finite element model (Fig. 2a) to alternatively simulate the effect of fluid injection and the 2011 earthquake slip in the area of the May 20, 2012 earthquake hypocentre (see the Methods section for details). The model comprised three horizontal strata and no faults. The model was biphasic with a homogeneous and continuous solid skeleton that represented the rock mass and pores that were filled with water. The equivalent geomechanical properties are reported in Table 1.

**Table 1:** Elastic, hydraulic and state parameters that were adopted in the numerical model.

Parameter	Name	Symbol	Value		
			Layer 1	Layer 2	Layer 3
Elastic parameters	Young’s modulus	E (Pa)	4 × 10^9^	1 × 10^10^	9 × 10^10^
	Poisson’s ratio	*ν*	0.23	0.25	0.2
Hydraulic parameters	Permeability	k (m^2^)	5 × 10^−12^	1 × 10^−15^	9 × 10^−15^
	Porosity	n	0.05	0.05	0.05
State parameters	Density	ρ (kg/m^3^)	2200	2350	2700

Different loads and solving phases were applied to the meshed geometry in Fig. 2a to simulate the stress changes from injection activities (hereinafter called the DIFF model) and the 2011 Mw 4.5 earthquake (hereinafter called the EQK model).

The DIFF model simulated fluid injection at the Cavone 14 well and computed the stress changes in the surrounding medium. The model geometry (Fig. 2a) crossed the Cavone 14 well and the 2012 earthquake hypocentre area (Supplementary Figure S2); this model is axisymmetric, with the cylindrical symmetry axis corresponding to the Cavone 14 well (the left side of the model in Fig. 2a and Supplementary Figure S2). The injection occurred approximately 3335 m below the surface (Fig. 2a and b). First, the initial stress and pore pressure conditions were established by applying a gravity load. The modelled pore pressure distribution at the end of this phase linearly increased with depth, which matched the *in situ* measurements at the Cavone 14 well^43^ (Supplementary Figure S3). Then, injection via the Cavone 14 well was simulated by applying a constant point flux of 1.28 × 10^5^ m^3^/yr over 20 years (Fig. 2b). The discharge corresponded to the mean volume of water that was reinjected at the Cavone 14 well since 1992 (Supplementary Figure S1).

The EQK model simulated the stress changes from the earthquake on July 17, 2011. Plane-strain analyses are typical when modelling thrust faults of different sizes^48, 49^ and in studies of fault reactivation and induced seismicity^50^. In our case, the perturbing earthquake was very small (Mw 4.5), which enabled us to adopt a plane-strain approximation. The fault was modelled using a contact interface with no friction, dipping 24° and extending approximately 900 m (red line in Fig. 2c). The modelled cross section was oriented perpendicularly to the fault’s strike (approximately 114°; EQK model in Supplementary Figure S2). This assumption was acceptable because the 2011 and 2012 events belonged to the same tectonic structures and had very similar strike (+/−10°) in the time-domain moment tensor solutions^51^. The slip was simulated by applying loads at the upper and lower edges of the fault (Fig. 2c). These loads had the same magnitude and direction but opposite effects. First, the initial distributions of stress and pore pressure (equal to the DIFF model) were established by applying the gravity load. Then, the 2011 Mw 4.5 earthquake was simulated in the coseismic and postseismic phases. In the coseismic phase, the loads were applied to the upper and lower faces of the interface (red line in Fig. 2c). The magnitudes of the loads were selected to create a maximum slip of approximately 7–8 cm along the fault plane (Supplementary Figure S4). In the postseismic phase, no further slip was applied to the fault plane; thus, only the pore pressure re-equilibrium from the coseismic pressure changes and the relative stress changes was calculated over 308 days prior to the 2012 mainshock (i.e., from July 17, 2011 to May 20, 2012).

### Pore pressure and Coulomb stress changes

We calculated the ΔCFS values for both the DIFF and EQK models (see the details in the Methods section). The ΔCFS was calculated on preferential thrust planes that struck 114° and dipped 40° counterclockwise with respect to the horizontal according to the mean retrieved dip and strike angles of the fault that was responsible for the event on May 20, 2012^40, 48, 52^. We assumed a positive sign convention for the tension and a negative convention for the compression in the model. We plotted the results in the entire domains of the DIFF and EQK models to consider uncertainty in the location of the fault, noting that the fluid pressures only reduced the normal stresses on the potential slip or rupture plane^53^.

**Figure 3:**
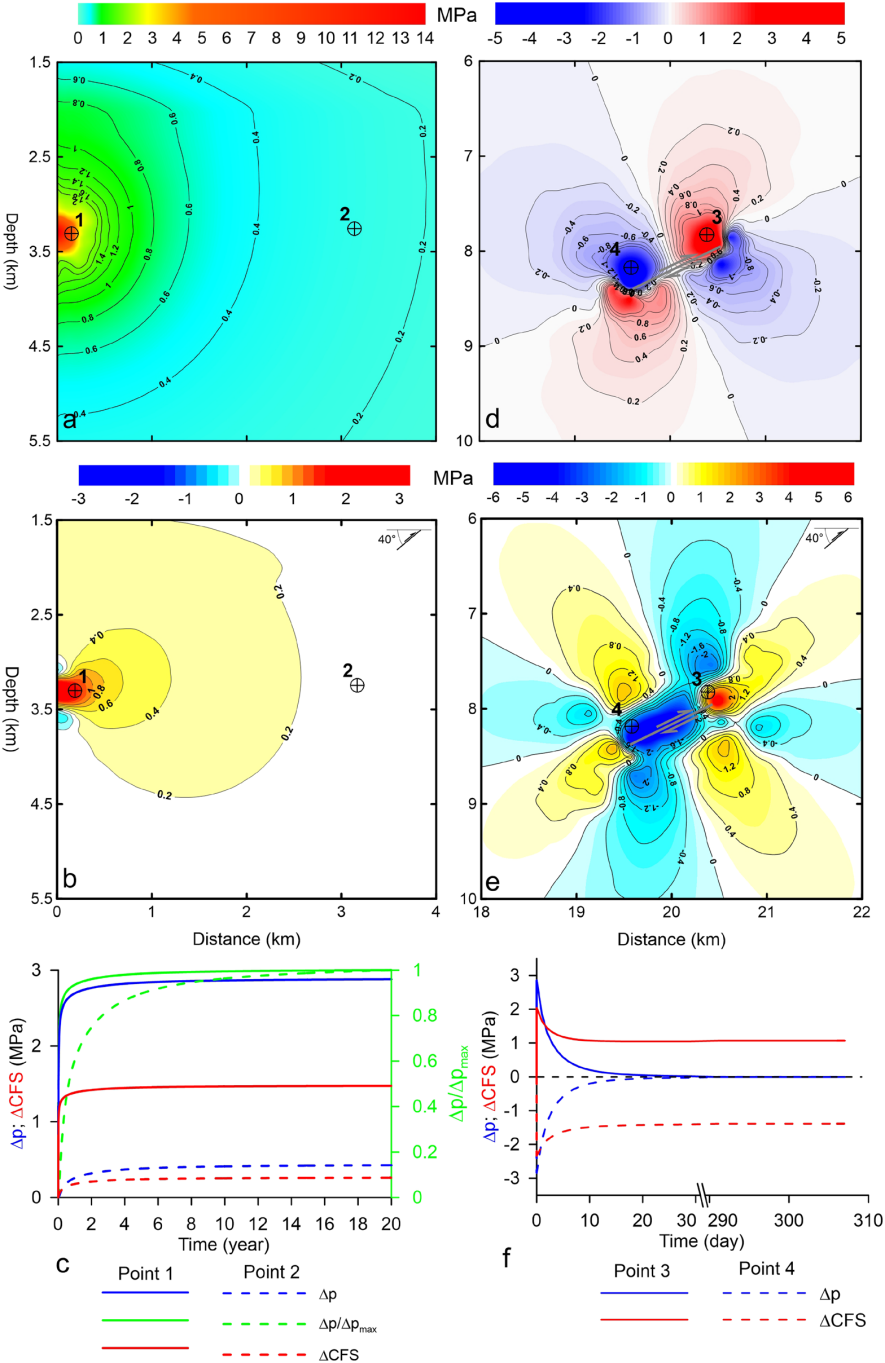
(**a**) Fluid overpressures after 20 years of injection at the Cavone 14 well (DIFF model; panel b in Fig. 2). (**b**) Coulomb stress changes after 20 years of injection at the Cavone 14 well. (**c**) Temporal evolution of the overpressures (Δp), overpressure ratio (Δ_p_/Δ_pmax_) and ΔCFS at points 1 and 2 in panels a and b. (**d**) Coseismic fluid overpressures and underpressures immediately after the simulated Mw 4.5 earthquake (EQK model; panel c in Fig. 2). (**e**) Coulomb stress changes ten months after the July 17, 2011 Mw 4.5 earthquake. (**f**) Temporal evolution of the overpressures (Δp) and ΔCFS at points 3 and 4 in panels d and e. The figures were created with Surfer® and Grapher® from Golden Software, LLC (www.goldensoftware.com). For context, the relative positions of the blown-up illustrations in (**a**), (**b**), (**d**) and (**e**) from Figs 3 and 4 are given in Supplementary Figures S5–S7.

In the DIFF model, the 20-year injection increased the pore pressure. The overpressures were approximately 14 MPa at the injection point (Fig. 3a). Then, these pressures rapidly decreased as the distance from the injection well increased because of the loss of the hydraulic head (Supplementary Figure S5). At the injection point (point 1 in Fig. 3a), the overpressures rapidly grew (solid blue line in Fig. 3c). Indeed, the maximum overpressure was reached approximately 4 years after the start of the injection, i.e., Δp/Δp_max_ = 1 (solid green line in Fig. 3c). At 3 km from the injection well (point 2 in Fig. 3a), the magnitude of the overpressure was lower because of the loss of the hydraulic head (blue dashed line in Fig. 3c). The delay from fluid diffusion caused the pore pressure to increase gradually rather than spiking. Moreover, a nearly constant overpressure occurred 18 years after the start of the injection (Δp/Δp_max_ = 1; green dashed line in Fig. 3c). At a greater distance, the overpressures were even smaller but continued to increase with time, given a constant injection rate (Supplementary Figure S5).

The overpressures and the resulting fluid diffusion modified the stress field and caused the Coulomb stress to change. A ΔCFS peak of approximately 3 MPa was reached at the injection point after 20 years (Fig. 3b). Then, a net decrease occurred at a certain distance (Supplementary Figure S6) according to the overpressure decay. Stress changes less than 0.2 MPa were observed 3 km from the Cavone 14 well (point 2 in Fig. 3b).

Near the injection location (point 1 in Fig. 3b), a ΔCFS peak of approximately 1.5 MPa was observed approximately four years after the start of the injection (solid red line in Fig. 3c and Supplementary Figure S6b). This increase was partially caused by the rapid increase in the shear stress from the elastic deformation of the solid skeleton (positive Δτ in Supplementary Figure S6b) but primarily by the pore pressure increase, which reduced the normal stresses on the fault plane (positive Δσ′ in Supplementary Figure S6b). At greater distances (point 5 in Supplementary Figure S6a), the ΔCFS increase was initially governed by the elastic deformation of the solid skeleton. Indeed, both the shear stresses and normal stresses increased (positive Δτ and negative Δσ′ in Supplementary Figure S6c). After approximately five years, the normal stresses began to decrease because of the arrival of the pore pressure pulse, i.e., Δσ′ began to increase, becoming positive after approximately 12 years. The computed ΔCFS values were transient and continued to increase because of the delay in the propagation of the pressure waves.

In the EQK model, the coseismic pattern of compression and dilation in the medium created a “bulls-eye”-shaped distribution of underpressures and overpressures, reaching approximately ± 5 MPa close to the fault (Fig. 3d). This pressure distribution was transient: the poroelastic effect prevailed initially, and mechanical deformation persisted over time. Indeed, the overpressures at points 3 and 4 (solid and dashed blue lines in Fig. 3f) reached nearly ± 3 MPa in the coseismic phase and later dissipated as fluid diffusion led to the re-establishment of hydrostatic conditions during the postseismic period, or after approximately 30 days. The required time to reach hydrostatic equilibrium depends on the hydraulic properties of the medium. In our case, the pore pressure distribution after 308 days (i.e., on the day of the May 20, 2012 event) corresponded to the initial hydrostatic pressure.

The ΔCFS in the EQK model at the time of the earthquake on May 20, 2012 exhibited positive and negative lobes close to the fault (Fig. 3e) with local values around ± 6 MPa. The stress perturbation became negligible (ΔCFS < ± 0.2 MPa) approximately 2 km from the plane (Supplementary Figure S7). This ΔCFS pattern was produced by the elastic stress change from only coseismic slip. The coseismic excess pore pressures with respect to the hydrostatic load were transient and rapidly dissipated. Indeed, the overpressures affected the ΔCFS during the first 30 days after the earthquake (solid and dashed red lines in Fig. 3f) but were negligible during the May 20, 2012 earthquake. However, the medium deformation was assumed to be permanent, and the mechanical stress changes persisted one year after the earthquake.

**Figure 4:**
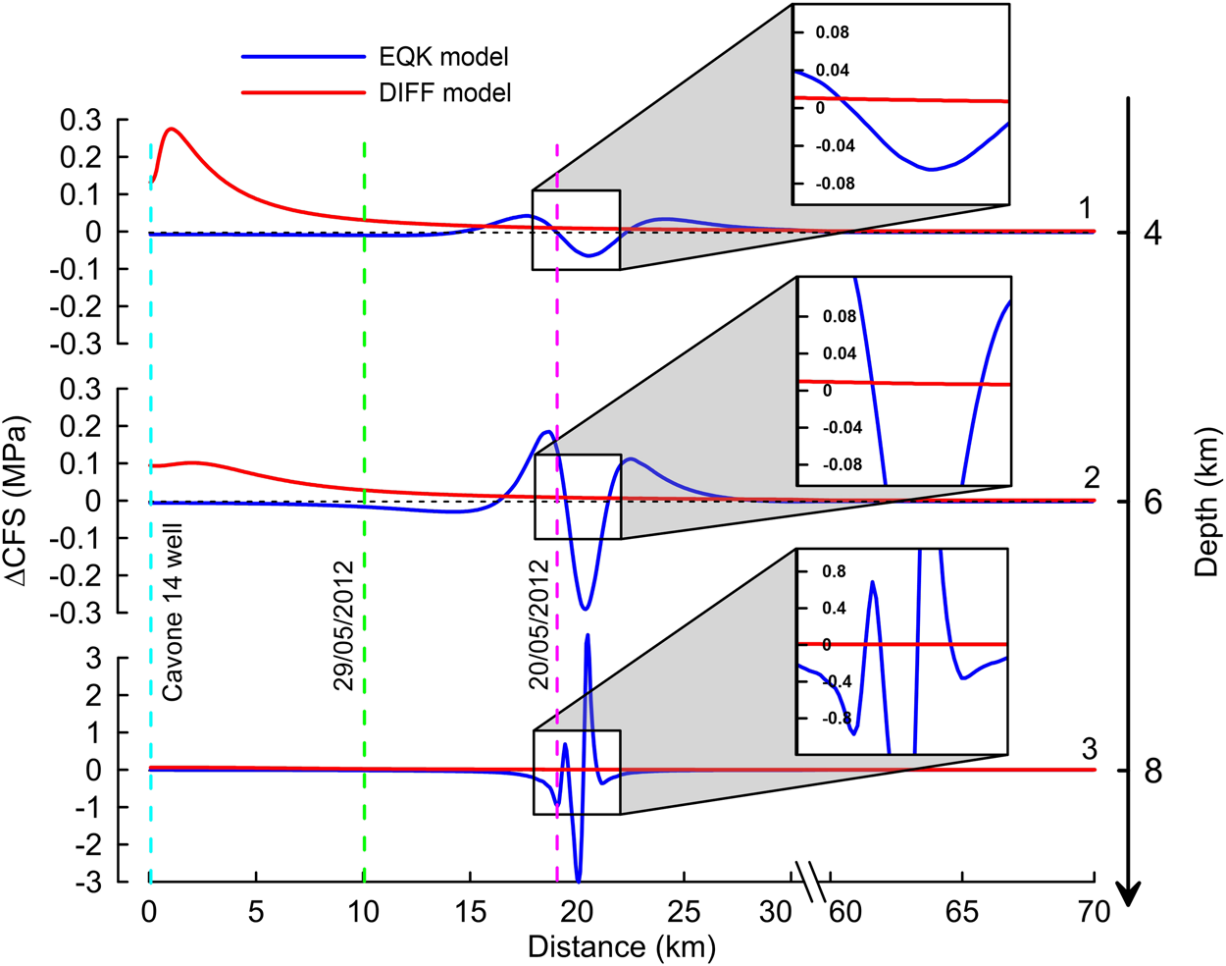
ΔCFS comparison between the DIFF and EQK models along horizontal sections 1, 2, and 3 in Fig. 2a. This figure was created with Grapher® from Golden Software, LLC (www.goldensoftware.com).

We compared the computed ΔCFS distributions from the DIFF and EQK models for the event on May 20, 2012, i.e., 20 years after the injection began at the Cavone 14 well, and the computed values 308 days after the earthquake on July 17, 2011. Given the difference between the numerical approximations in the DIFF and EQK models, the comparison was only performed at the location of the May 20, 2012 event, i.e., approximately 20 km from the left side of the model, where the DIFF and EQK models intersected (dashed violet line in Fig. 4 and Supplementary Figure S2). We drew ΔCFS profiles along three horizontal sections at depths of 4, 6, and 8 km (sections 1, 2, and 3 in Fig. 2a). These sections crossed the area of the event on May 20, 2012, which was located at a depth of approximately 6 km and was 19 km from the Cavone 14 well (yellow star in Fig. 2a and violet dashed line in Fig. 4)^54^. The maximum ΔCFS from the DIFF model was approximately 0.3 MPa near the injection point and decreased to less than 0.01 MPa at a distance of approximately 20 km (profiles 1, 2 and 3 in Fig. 4). The Mw 4.5 earthquake on July 17, 2011 produced positive and negative ΔCFS values; i.e., the stress increased in some areas and decreased in others. The stress perturbation extended more than 5 km from the fault. The positive ΔCFS values were greater than 3 MPa close to the fault (profile 3 in Fig. 4) and approximately 0.04 MPa at a distance of 4 km (e.g., see profile 1). The peak ΔCFS was 0.2 MPa at the hypocentre of the May 20^th^ event (intersection of the violet dashed line and profile 2), i.e., more than ten times larger than the ΔCFS from fluid injection. Thus, the relative effects of the two phenomena depended on the distance between the mainshock and the oilfield or the reference earthquake.

## Discussion

The ΔCFS from the injection in the hypocentre area of the May 20^th^ event was approximately 5–10% of the ΔCFS from the 2011 earthquake (Fig. 4). Thus, the injection at Cavone 14 contributed much less to advancing the Ferrara thrust toward failure than a natural earthquake. Therefore, the triggering of the Mw 5.9 earthquake on May 20, 2012 was likely caused by a natural stress increase rather than anthropogenic activities.

It is worth discussing the significance of the results regarding the assumptions that were used to develop the model. We assumed a continuous, constant, 20-year injection flux. In reality, the injection activity was discontinuous and characterized by a quasi-periodic cycle of three days of injection and two days off. The associated cessation of injection produced a sudden pore pressure drop in the borehole. However, at the distances of interest (i.e., more than 5 km), this cyclic effect was delayed by several tens of days^55^ and smoothed by the slow diffusion of the pressure field away from the borehole. In fact, the peak pressure often occurred in the far field when pressurization at the injection point had terminated^1, 56^.

For simplicity, we also modelled the injection without considering the extraction of crude oil in nearby wells. Experimental and geological considerations support such an assumption. According to Segall^57^, the stresses from oilfield depletion cause reverse faulting above and below the reservoir, while normal faulting occurs along the flanks of the oilfield. In our case, the structure that was responsible for the May 20^th^ event was located more than 10 km from the Cavone oilfield (Fig. 1a) and in an area where oilfield depletion may have promoted normal faulting instead of thrusting; thus, the effect of reservoir depletion was likely negligible in this case^3, 44, 45^. The presence of a low-permeability layer between production and injection wells and the absence of interactions, at least for intermediate periods, between production and injection wells^43^ suggest that the oil reservoir and the deep aquifer were hydraulically disconnected. Thus, we disregarded the hypothesis of using a net injection rate (i.e., the difference between the total injected and total produced quantities). Moreover, neglecting the production wells led to an overestimation of the pore pressure in the medium. Thus, the modelled changes in the anthropogenic stress defined the worst-case scenario, which can be compared with natural stress changes from the 2011 earthquake.

We assumed that the medium was fully saturated (Skempton coefficient B = 1). This hypothesis is acceptable because fluids at seismogenic depths can be considered water or similar to water^28^. Nonetheless, smaller values of B can be found, especially for low confining pressures (i.e., close to the surface) and for saturated sedimentary soils. For rocks, B values lower than one are commonly associated with the presence of a small percentage of gas bubbles, which fill voids that comprise the unconnected porosity of the medium or are trapped inside fluids that fill the interconnected pores. These bubbles modify neither the fluid pressure distribution with depth, which remains hydrostatic, nor the fluid diffusion, which continues to obey Darcy’s law. However, a mechanical compression of a porous medium with B less than one produces lower overpressures. Indeed, some of the volumetric strain is accommodated by the compression of the gas bubbles. Consequently, the calculated overpressures and the ΔCFS in the DIFF model represented an upper boundary with respect to the expected values. In the EQK model, the ΔCFS at the time of the 2012 event was only caused by the permanent elastic deformation of the medium. Thus, a change in the overpressure amplitude did not affect the ΔCFS pattern in Fig. 3e.

The results of this model were dependent on the assumed permeability. Indeed, the permeability for Layer 3 (Table 1) was back-calculated by fitting the experimental data of a pumping test in the study area (see the Methods section for details). However, the calibrated value was representative of depths of 3–4 km, while the confining pressure at seismogenic depths (6–10 km) was high and fractures tended to close; thus, we could expect a lower permeability^58^. However, low permeability means a high loss of the hydraulic head; therefore, the calculated overpressures and ΔCFS for the DIFF model at the 2012 earthquake hypocentre overestimated the real values. For the EQK model, a change in the permeability could have affected the diffusion process but did not modify the ΔCFS pattern at the time of the 2012 event because of the permanent elastic deformation of the medium.

Our poroelastic model does not include long-range effects such as channel flow, near-borehole fracturing effects, or dynamic effects^7, 8, 59^. This choice is justified because the relatively small pore pressures at depth during the operative injection^43^ (approximately 14 MPa in Figure S8b) did not induce significant hydrofracturing or fracturing close to the borehole (see the Methods section for details). Significant long-range effects were also averted because the thrust system in the study area consisted of several thrusts and back thrusts that were not connected. The unmodelled effects were represented by the difference between the experimental and modelled curves for the calibration of the finite element model (Figure S8b; RMSE of approximately 0.85 MPa).

Finally, we neglected the effect of the temperature gradient from the injected water at depth; however, the temperature gradient in the study area produced negligible stress changes in the surrounding medium^45^.

Another important issue is determining whether the Mw 5.7 earthquake on May 29, 2012 was advanced toward failure by the activities in the Cavone oilfield. Although this topic is not the focus of this study, some findings can be noted. The earthquake occurred along the SE edge of the Cavone oilfield, approximately 10 km from the Cavone 14 well (Fig. 1a). The injection-induced ΔCFS (approximately 0.05 MPa in Fig. 4) was only a fraction of the approximately 0.6 MPa of pressure from the May 20^th^ event^40^. The rupture of the Mirandola thrust (the May 29^th^ event) was likely triggered by the additional stress load that was redistributed after the Ferrara thrust broke (the May 20^th^ event). However, the fault approached failure because of the 0.05 MPa of force rather than only natural stresses; thus, the May 29^th^ rupture area could have increased under the same pre-existing tectonic stresses. The trigger could be considered tectonic, but fracture propagation may have been facilitated by the increased pore pressure, resulting in a slightly larger rupture area.

The ΔCFS distribution in the EQK model was affected by uncertainties because this result was dependent on the relative locations of the 2011 and 2012 earthquakes, their fault geometries, and the assumed friction coefficient. In our case, the uncertainties of the hypocentres were estimated to be approximately 5 km. The uncertainties in the location of a moderate-size earthquake depend on the geometry and quality of the seismic network and knowledge of the subsurface structure. However, such uncertainties mainly affect the relative locations of ΔCFS peaks but not their magnitudes. Consequently, we can still assume that the effect of anthropogenic activities at the Cavone oilfield was negligible compared to natural stress changes from low-magnitude earthquakes.

A variation of 10° in the strike and rake yielded insignificant changes in the stress field. The most sensitive parameter was the fault dip. We evaluated that a change of 10° in the target fault dip (i.e., 30°, 40° and 50°) yielded a change up to 20% in the ΔCFS for the DIFF model, leaving the sign unchanged (Supplementary Figure S9a and b). For the EQK model, the ΔCFS pattern slightly rotated, thus producing variations in the sign and amplitude of the ΔCFS, especially at point 3, which was very close to the fault (Supplementary Figure S10a). However, the adopted fault dip (40°) was well constrained^40, 60^, and therefore, the conclusions of our study remain unchanged.

A change of 0.1 (i.e., μ = 0.3, 0.4 and 0.5) in the friction coefficient did not affect the sign of the ΔCFS for both the DIFF and EQK models, while the ΔCFS amplitude varied by less than 25% (Supplementary Figures S9c and d and S10b).

The ability to trigger an upcoming earthquake depends on the criticality of the fault, namely, how close the fault is to failure. Therefore, we must appreciate that the size of Coulomb stress changes does not necessarily exclude the importance of fluid injection on future earthquakes.

The main result of our analysis suggests that moderate-magnitude earthquakes perturbed the stress levels of nearby seismogenic faults more so than anthropogenic activities. Stress perturbations are based on the magnitude of the perturbing earthquake, the distance between the shock and the target fault, and the fault geometry and kinematics. This conclusion can be generalized and suggests that stresses and pressures must be accurately modelled when injection activities occur in seismogenic areas. However, evaluating only the amplitude of the anthropogenic ΔCFS cannot exclude the importance of fluid injection on future earthquakes because the ability to trigger an upcoming earthquake depends on how close the fault is to failure, which is unknown. Consequently, high-resolution seismic networks must be deployed and maintained to reduce the uncertainties that are associated with earthquake locations and mechanisms. Only knowledge regarding the relative effects of moderate-size earthquakes and pore pressure variations can be used to assess seismic hazard changes in areas that surround oilfields. Additionally, intervention protocols, which can sometimes fail, benefit from these prior evaluations that consider the locations, magnitudes, and geometries of the different earthquake scenarios. The modelling complexity is case dependent and based on the geometrical, mechanical, and hydraulic characteristics of the area. Given the large potential differences, modelling must be re-performed when earthquakes that differ from typical scenarios occur during oilfield activities.

Our model showed that the injection activities at the Cavone oilfield had a negligible influence on triggering the Emilia Romagna seismic sequence in 2012. Thus, the seismic hazard from the Ferrara thrust was not significantly increased by activities in the Cavone oilfield, at least when compared to natural stress perturbations from a previous tectonic earthquake. However, this result presumes that the assumptions of the model were correct.

The debate regarding the anthropogenic triggering of large earthquakes and their effect on society continues. Regardless of the seismic activity in a region, the seismic risk must be quantified and managed in populated areas because of subsurface exploitation. Both numerical experiments and field tests can describe the physics of anthropogenic earthquakes^19, 56, 61^. However, most models predict changes in the rate of recurrence and not the rate itself, therefore, seismic hazard changes must be quantified on a parametric basis in terms of varying seismicity rates^9^. Forecasting the maximum magnitude of a potential trigger based on the calculated rate of change is difficult. Nevertheless, strategies that involve a given threshold (e.g., the “traffic light system”) have been implemented to manage the risk from enhanced geothermal systems^5, 20, 62–64^. These strategies are often empirical and based on the real-time seismic monitoring of the maximum magnitude and (sometimes) rate. If the magnitude of the triggered earthquake surpasses a given threshold, injection activities are reduced or shut down. However, these strategies are only effective during the early stages of the injection process. In fact, the effectiveness of reducing the injection rate at the wellhead diminishes with time and space as the pore pressure front migrates away from the injection point^1, 13, 56^. This complexity illustrates one of the advantages of the physical modelling approach that is described herein, which can be implemented in real time to improve the current traffic light approach.

## Methods

### Relocation and slip estimation of the July 17, 2011 Mw 4.5 earthquake

On July 17, 2011, an Mw 4.5 earthquake occurred on the Po plain. The earthquake was felt extensively in the same area as the 2012 Mw 5.9 mainshock. The location of the 2011 earthquake was affected by significant uncertainty (up to 20 km) because the Po plain has relatively few seismic stations (because of the high level of seismic noise) and is characterized by large heterogeneities in velocity models. However, the macroseismic intensity patterns of the earthquakes that occurred on July 17, 2011 (Mw 4.5) and May 20, 2012 (Mw 5.9) were similar, with maxima in identical or nearby villages and intensities that decreased similarly^65^. Moreover, the focal mechanisms were similar. These similarities suggest that the hypocentres of the Mw 4.5 and Mw 5.9 events were spatially proximate. Therefore, we modelled the 2011 Mw 4.5 earthquake as a potential stress perturbation of the 2012 earthquake. We simulated the slip of an approximately 900-m-wide fault plane by using a plane strain approximation. Additionally, multiple forces (edge loads) were applied to the thrust fault, resulting in a coseismic slip of approximately 7–8 cm.

The mean slip of the Mw 4.5 earthquake on July 17, 2011 was estimated by using equation (2)^66^:

2 $${M}_{0}=G\cdot S\langle u\rangle $$

where M_0_ is the seismic moment, G is the shear modulus, S is the fault plane surface, and $\langle u\rangle$ is the mean slip on the fault plane. We assumed a fault area of 2–2.5 km^2^, which corresponds to a length of 2.2–2.8 km and a width of 900 m. The adopted shear modulus of G = 37.5 GPa was consistent with the stiffness parameters of layer 3 in Table 1. The seismic moment was estimated by using equation (3)^67^:

3 $${M}_{w}=\frac{2}{3}\cdot ({\mathrm{log}}_{10}{M}_{0}-9.81)$$

where Mw corresponds to the moment magnitude of the earthquake on July 17, 2011.

### Finite element modelling

The finite element model grid extended 70 km from the injection location and 20 km deep. The mesh comprised eight-node, isoparametric, arbitrary quadrilateral elements (2498 elements). The grid had an element size of approximately 0.2 km close to the injection point and the fault discontinuity (Fig. 2b and c). Then, the element size increased to approximately 1 km near the lateral and bottom edges of the domain.

The mechanical boundary conditions consisted of orthogonal fixities at the bottom and sides of both models. For the hydraulic boundary conditions, an atmospheric pressure condition was set as the pore pressure at the surface, while the lower edge was assumed to be impermeable in both the DIFF and EQK models. In the DIFF model, a prescribed nodal mass flow rate was applied to the left side at a depth of 3335 m to simulate the water injection (Fig. 2b). The left edge was also set to be impermeable because of the axial symmetry condition. The right edge of the model was assigned a hydrostatic pore pressure condition because this edge was located far from the injection point. However, we found that the hydrostatic boundary condition had a negligible effect on the pore pressure pattern.

In the EQK model, the fault was modelled with a straight, 900-m-long contact interface with no friction, where the upper and lower mesh elements were allowed to slip. The slip between the upper and lower mesh elements was simulated by applying uniform edge loads along the top and bottom edges of the contact interface (Fig. 2c). The edge loads were used to simulate only the coseismic slip, and no further external loads were applied during the postseismic phase. The magnitude of the edge loads was calibrated to reproduce a mean relative displacement of approximately 7–8 cm (Supplementary Figure S4)

The pore pressures on both sides were assumed hydrostatic. Additionally, the pore pressure perturbation from the simulated slip was localized around the dislocated fault and was not affected by the hydrostatic boundary condition.

The developed finite element model solves linear-poroelastic relationships^68^ by using the commercial software MSC Marc 2015 ^69^. Specifically, the presence of fluids within a medium is modelled by introducing two quantities: the pore fluid pressure *p* and the mass content *m* of diffusing fluids per unit volume of porous solid. For a poroelastic medium, the constitutive equations are as follows^70^:

4 $$2G{\varepsilon }_{ij}={\sigma }_{ij}-\frac{\nu }{1-\nu }{\sigma }_{kk}{\delta }_{ij}+\frac{(1+2\nu )\alpha }{1+\nu }p{\delta }_{ij}$$

5 $${\rm{\Delta }}m=m-{m}_{0}=\frac{(1-2\nu )\alpha \rho }{2G(1+\nu )}\cdot [{\sigma }_{kk}+\frac{3}{B}p]$$

Equation (4) relates the stresses and strains in a poroelastic medium, while equation (5) relates the change in fluid mass per unit volume to both the mean stress (*σ*_*kk*_) and pore pressure changes. *G* and *ν* are the drained shear modulus and Poisson’s ratio, respectively; *ε*_*ij*_ and *σ*_*ij*_ are the strain and stress tensor components, respectively; *p* is the pore pressure; *B* is the Skempton coefficient; *ρ* is the fluid density; *m* is the fluid mass content; *m*_0_ is the fluid mass content for a reference state, which is the product of the fluid density *ρ* and the volume fraction of the pore space (i.e., the porosity *n*); *δ*_*ij*_ is the Kronecker delta; and *α* is the Biot coefficient.

The fluid flow *q* is governed by Darcy’s law (equation 6), where *k* is the intrinsic permeability of the medium, *η* is the fluid viscosity, *ρ* is the fluid density, and *p* is the pore pressure:

6 $$q=-\rho \frac{k}{\eta }\nabla p$$

We assume that the fluid is water with a density *ρ* = 1000 kg/m^3^, viscosity *η* = 0.001 Pa·s, and bulk modulus *K* = 2.2 × 10^9^ Pa. Darcy’s law is only applicable to laminar flow. The flow motion is empirically laminar at flow rates less than approximately 0.5 × 10^5^ m^3^/day, and becomes turbulent at flow rates greater than 10^5^–1.5 × 10^5^ m^3^/day^71^. At the Cavone 14 well, the maximum injection rate of approximately 5.26 × 10^2^ m^3^/day (Figure S1) satisfied the laminar flow condition.

We assumed that the solid skeleton in the current formulation was incompressible (*α* = 1) and that the pores were fully saturated (*B* = 1). The other material constants that had to be determined were the drained shear modulus *G*, the drained Poisson’s ratio *ν*, the permeability *k*, the porosity *n* and the density *ρ*.

The geometry of the model outlined the main characteristics of the subsurface in the investigated area (Fig. 1c). Several thrusts that were buried below the Quaternary sedimentary layers dissected carbonatic and arenaceous lithologies and Pre-Pliocene and Post-Pliocene successions^33, 72^, forming a complex joint system in which the stiffness, strength, and hydraulic properties of the material depend on the stress field^73^. Moreover, the behaviour of the geomaterial is strictly dependent on the intrinsic and state features of the materials, such as the local geometric and rheological anisotropies, joint density and orientation, and previous stress paths, which significantly influence the mechanical and hydraulic properties at the scale of tens of metres.

The selection of model parameters depends on the model size. We adopted an equivalent continuum approach^74^ in our kilometre-scale model. Following this approach, we assumed that the rock strata were isotropic, homogeneous and continuous and derived the equivalent parameters from the geomechanical properties of both intact rock and joints.

From a hydraulic perspective, the horizontal permeability is much greater than the vertical permeability because of the presence of horizontal shear stresses and fractures that are typically oriented parallel to the shear. Therefore, we emphasized the hydraulic connection between the injection point and the May 20^th^ hypocentre area by assuming a single permeability value that approximately represented the horizontal flow. We considered three strata in both the DIFF and EQK models: an upper Quaternary layer (Layer 1), a middle Pliocene-Miocene layer (Layer 2), and a lower Jurassic- Triassic layer (Layer 3)^75^.

### Model calibration

The model parameters are listed in Table 1. The elastic and state parameters that we used were consistent with those that were employed in other models of the study area^48, 76^. The hydraulic parameters require further discussion because they affect the magnitude, distribution, and temporal variation in the pore pressures. According to rock samples and conductivity tests, the values of the intrinsic permeability and porosity were extremely heterogeneous, ranging from 1 to 1000 mD (milliDarcy) and from 0.5 to 20%, respectively^43, 44^. To properly calibrate the hydraulic parameters, we analysed the results of a pumping test that was performed at the Cavone 14 well in 2014.

The pumping test at the Cavone 14 well was performed from May 13, 2014 to June 16, 2014^43^. An injection test was established in three steps:

A. Injection fall-off at the Cavone 14 well (May 13, 2014).
B. Ninety-six hours of injection (May 23–27, 2014) with a constant discharge of 600 m^3^/h.
C. Injection closure for 480 h (May 27-June 14, 2014).

During the test, the pore pressure was measured with a memory gauge approximately 90 m above the bottomhole depth (Fig. 2b). The profiles of the static pore pressure (Supplementary Figure S3) and temperature were measured during the installation (May 16, 2014) and removal (June 16, 2014) of the memory gauge.

The interpretation of the experimental pore pressure profile (Supplementary Figure S8a) highlighted the following findings: (i) Over short periods, the absence of a mechanical skin at the wellbore suggested that the well operations did not damage the rock. A high-permeability zone was also detected that extended approximately 100 m from the well (less than the minimum dimension of the mesh). (ii) Over intermediate periods, a dual-porosity behaviour was observed. The fractured rock exhibited different matrix and fracture porosities, and the fractures governed the fluid flux. (iii) Over extended periods, the results reflected a partially confined reservoir. Then, we adopted a constant permeability value in our model that represented a uniform fault distribution inside the medium, which is typically used when modelling dual-porosity reservoirs^64^. Finally, we back-analysed the pumping test results by using the DIFF model.

The pore pressure that was measured at the memory gauge (Supplementary Figure S8a) was influenced by variations in the long wavelength pressure because of the injection fall-off during phase A. This variation was removed for a comparison with the numerical results (Supplementary Figure S8b).

We simulated the pumping test in three phases. During the first phase, hydrostatic conditions were established for the stresses and water pressures (Supplementary Figure S3) by applying a gravity load. During the second phase (corresponding to step B), the injection was simulated by using a constant discharge of 600 m^3^/h for 96 h. During the last phase (corresponding to step C), the injection was stopped, and the decrease in pore pressure was monitored for 480 h at the grid node that corresponded to the position of the memory gauge (Fig. 2b).

We varied the hydraulic parameters to reproduce the pore pressure trend that was measured by the memory gauge during steps B and C. The results, which were expressed via pore pressure increases based on the hydrostatic pressure (Supplementary Figure S8b), exhibited a good fit between the modelled and measured pressures (RMSE = 0.85 MPa). The calibrated hydraulic parameters are given in Table 1. The retrieved parameters fell within the experimental ranges of the intrinsic permeability and porosity and were typical of a semi-pervious aquifer^77^. A sensitivity analysis was conducted by varying the hydraulic and mechanical parameters that were adopted in the model. The elastic properties of all the layers and the hydraulic constants of layers 1 and 2 did not significantly affect the calculated pore pressure, and the results were more sensitive to the parameters of the layer that was directly involved in the injection. In fact, varying the hydraulic parameters by one order of magnitude in layer 3 significantly changed the magnitude and shape of the pore pressure curve. Thus, calibrating the poroelastic parameters by back-analysing the *in situ* data was a crucial step in our modelling approach.

### Calculation of the Coulomb stress change

The Coulomb stress changes were calculated at faults with a specified orientation of 40° from the horizontal. We assumed a reference system in which the x-axis, y–axis, and fault displacements were horizontal and the fault planes were vertical and parallel to the z-direction (Fig. 2a). Within this reference system, the stress on a plane at an angle ψ from the x-axis is given by equations (7) and (8)^23^:

7 $${\sigma }_{33}={\sigma }_{xx}\cdot {\sin }^{2}\psi -2{\sigma }_{xy}\cdot \,\sin \,\psi \,\cos \,\psi +{\sigma }_{yy}\cdot {\cos }^{2}\psi $$

8 $${\tau }_{13}=\frac{1}{2}({\sigma }_{yy}-{\sigma }_{xx})\cdot \,\sin \,2\psi +{\tau }_{xy}\cdot \,\cos \,2\psi $$

where τ_13_ is the shear stress (which is positive in the slip direction), and σ_33_ is the normal stress (tensile stress is positive) on the fault plane. The variables σ_xx_, σ_yy_, and τ_xy_ are the normal and shear stresses that were calculated by using our numerical models in the reference system in Fig. 2a.

Thus, the change in the Coulomb stress is given by equation (9) ^29, 30^:

9 $${\rm{\Delta }}CFS={\rm{\Delta }}{\tau }_{{13}}+\mu \cdot ({\rm{\Delta }}{\sigma }_{{33}}+{\rm{\Delta }}p)={\rm{\Delta }}{\tau }_{{13}}+\mu \cdot {\rm{\Delta }}{\sigma }_{{33}}^{^{\prime} }$$

where Δp is the pore pressure change that is computed by the model and Δσ′_33_ is the effective normal stress. We assume a positive sign convention for tension and a negative convention for compression in this model; thus, a positive ΔCFS indicates fault weakening.

Selecting a proper friction angle is not straightforward. Friction coefficients are highly variable in the field and laboratory. Values of 0.6–0.8 typically refer to undisturbed or reconstituted rock specimens that are tested in laboratories with confining stresses that are lower than crustal stresses^78^. These values can be effectively used to simulate the near-surface failure behaviour of rocky materials^79^.

In this study, the rock mass was not intact but was jointed and fractured. Additionally, this rock mass was subjected to high confining stresses. The presence of joints and high confining pressures substantially reduce the friction angle^80^. Values of 0.3–0.4 are typical of fault zones^81, 82^; therefore, we selected a friction coefficient of μ = 0.4.

However, the choice of a different friction coefficient did not alter the results of our study. In fact, we do not discuss the Coulomb stresses in terms of absolute values (which depend on the selected friction coefficient). Instead, we compare the Coulomb stress changes from two different phenomena. Consequently, choosing an appropriate friction coefficient is a second-order problem.

## Electronic supplementary material

Supplementary figures
