# Supplementary material for: Discriminating between natural and anthropogenic earthquakes: insights from the Emilia Romagna (Italy) 2012 seismic sequence
Source: Sci Rep. 2017 Mar 21;7:282. doi: 10.1038/s41598-017-00379-2 (PMC5428219; doi:10.1038/s41598-017-00379-2)
Supplement: Supplementary file 1 — Supplementary figures [file 41598_2017_379_MOESM1_ESM.pdf]

# Supplementary material for "Discriminating between natural and anthropogenic earthquakes: insights from the Emilia Romagna (Italy) 2012 seismic sequence"

**Matteo Albano[1*], Salvatore Barba[1+], Gabriele Tarabusi[1,2+], Michele Saroli[3,1+] and Salvatore Stramondo[1+]**

[1] Istituto Nazionale di Geofisica e Vulcanologia, Via di Vigna Murata 605, 00143 Roma, Italy

[2] Dept. of Physics and Earth Sciences, Ferrara University, Via Saragat 1, 44122 Ferrara, Italy

[3] Università di Cassino e del Lazio Meridionale, Via G. di Biasio 43, 03043 Cassino, Italy

*matteo.albano@ingv.it

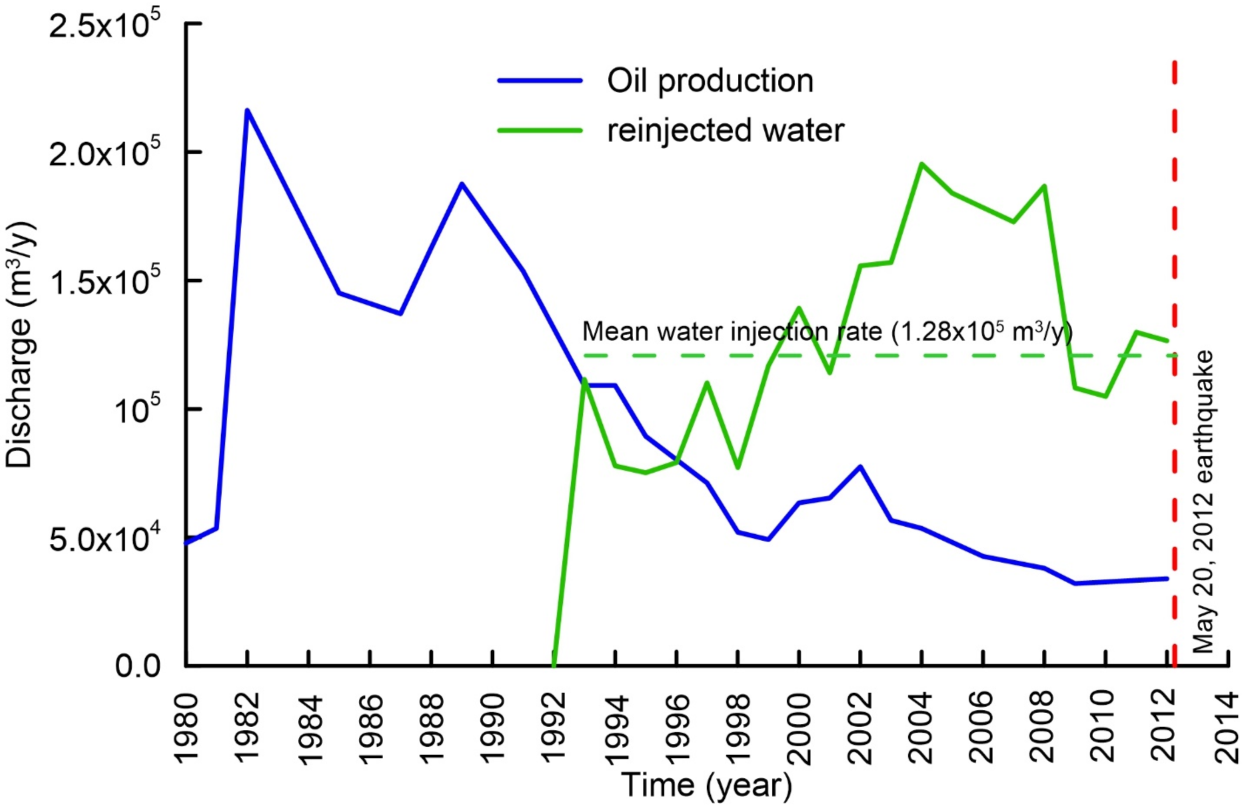


**Figure S1.** Yearly oil production and water reinjection at the Cavone oilfield[44]. This figure was created with Grapher® from Golden Software, LLC (www.goldensoftware.com).

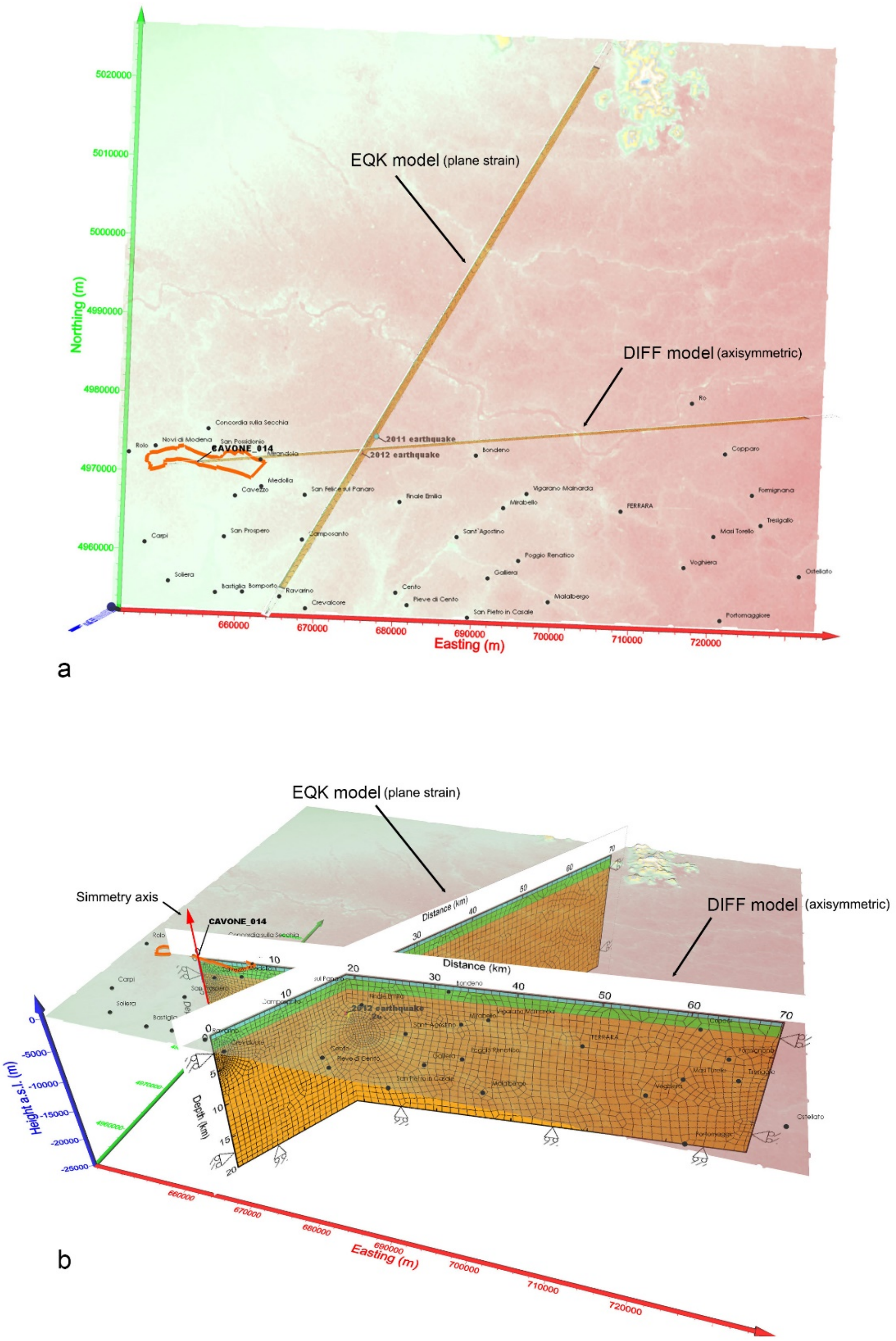


**Figure S2.** Location of the DIFF and EQK models in the study area. The two sections intersect at the May, 20 2012 hypocentre area. a) Plain view. b) 3D view. This figure was created with Voxler® from Golden Software, LLC (www.goldensoftware.com).

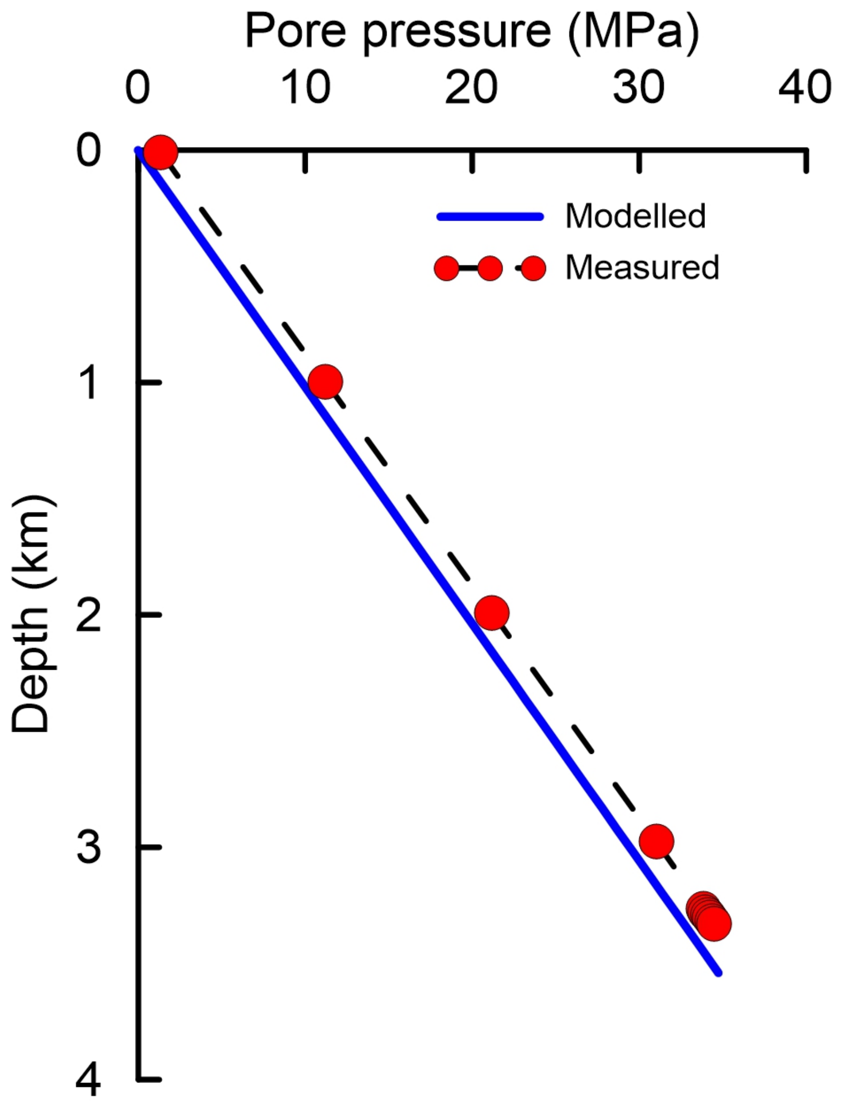


**Figure S3.** Measured[43] and modelled profiles of pore pressure with depth. This figure was created with Grapher® from Golden Software, LLC (www.goldensoftware.com).

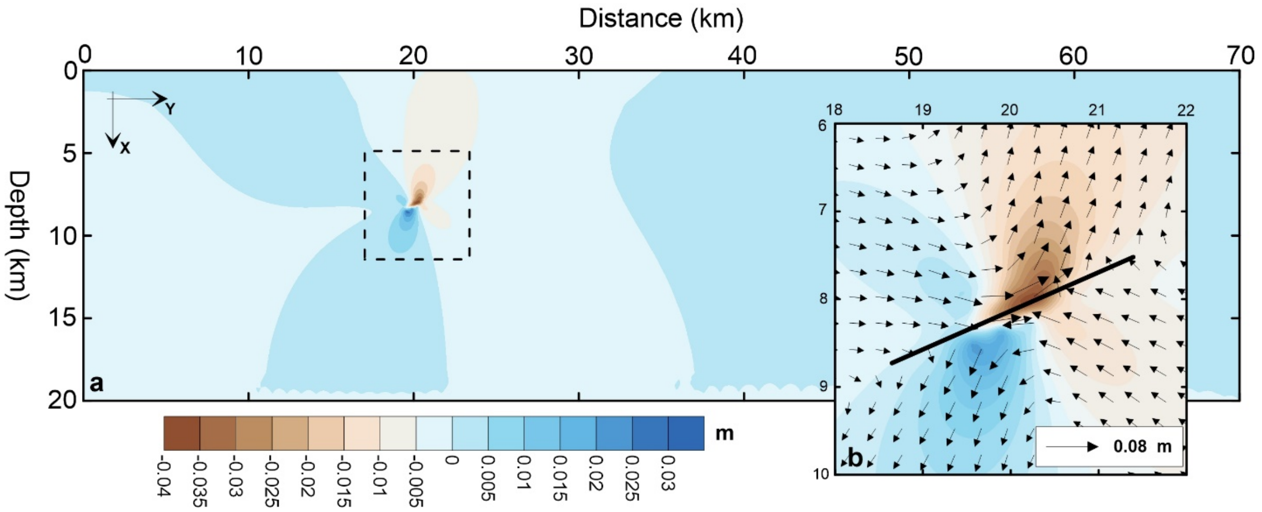


**Figure S4.** (a) Coseismic resultant displacement after simulating the slip of the July 17, 2011 Mw 4.5 earthquake. The black square refers to the detailed area in panel b. (b) Detailed slip at the simulated fault plane (black line). This figure was created with Surfer® from Golden Software, LLC (www.goldensoftware.com).

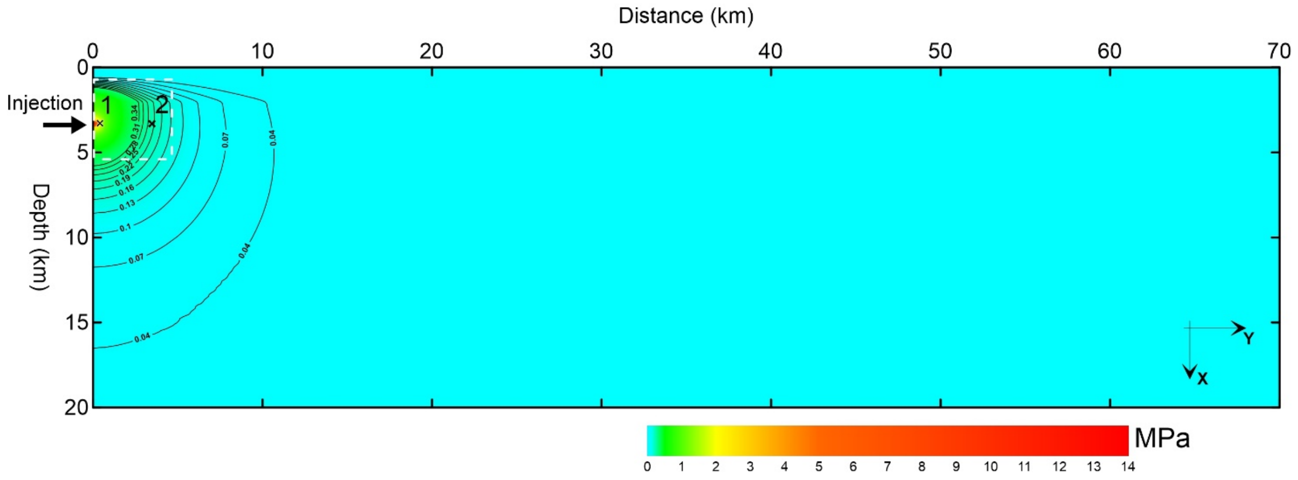


**Figure S5.** Distribution of the overpressures after 20 years of injection at the Cavone 14 well. The white box refers to the detailed area in Figure 3a. Points 1 and 2 refer to the overpressure histories in Figure 3b. This figure was created with Surfer® from Golden Software, LLC (www.goldensoftware.com).

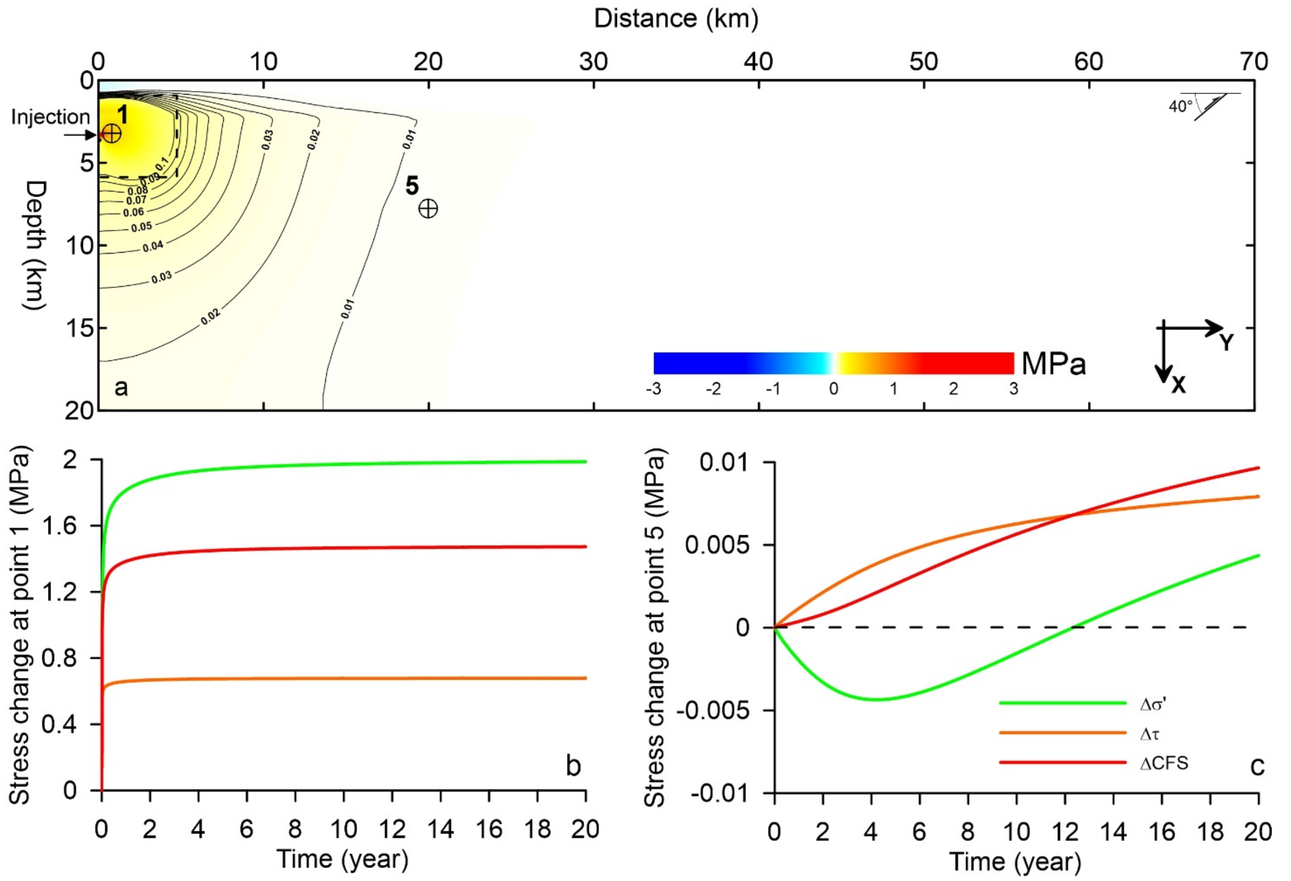


**Figure S6.** (a) Distribution of the ΔCFS after 20 years of injection at the Cavone 14 well. The white box refers to the detailed area in Figure 3b. (b) Temporal evolutions of the normal stress change (green line), shear stress change (orange line), and Coulomb stress change (red line) at point 1 in Panel a. (c) Temporal evolutions of the normal stress change (green line), shear stress change (orange line), and Coulomb stress change (red line) at point 5 in Panel a. This figure was created with Surfer® from Golden Software, LLC (www.goldensoftware.com).

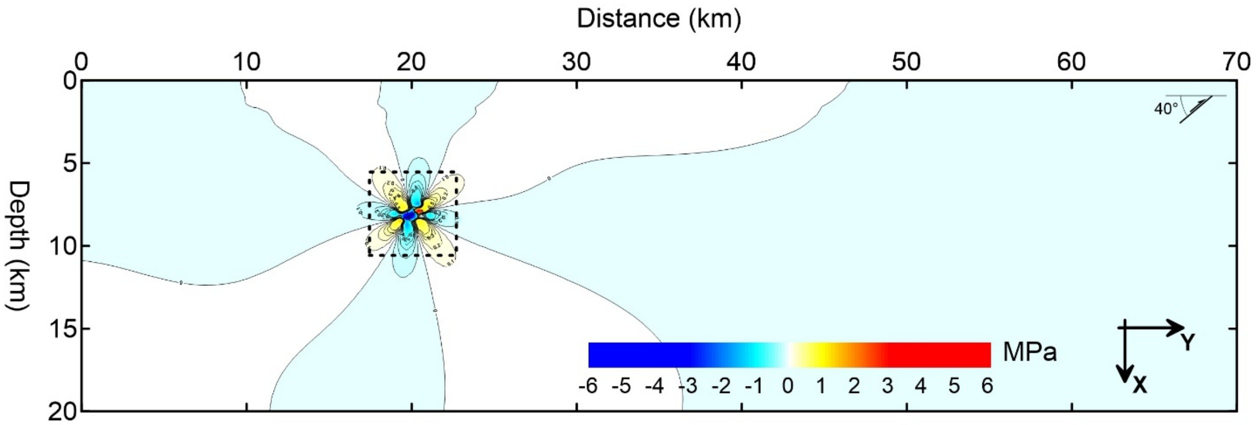


**Figure S7.** Distribution of the ΔCFS ten months after the July 17, 2011 Mw 4.5 earthquake. The dashed black square refers to the detailed area in Figure 4b. This figure was created with Surfer® from Golden Software, LLC (www.goldensoftware.com).

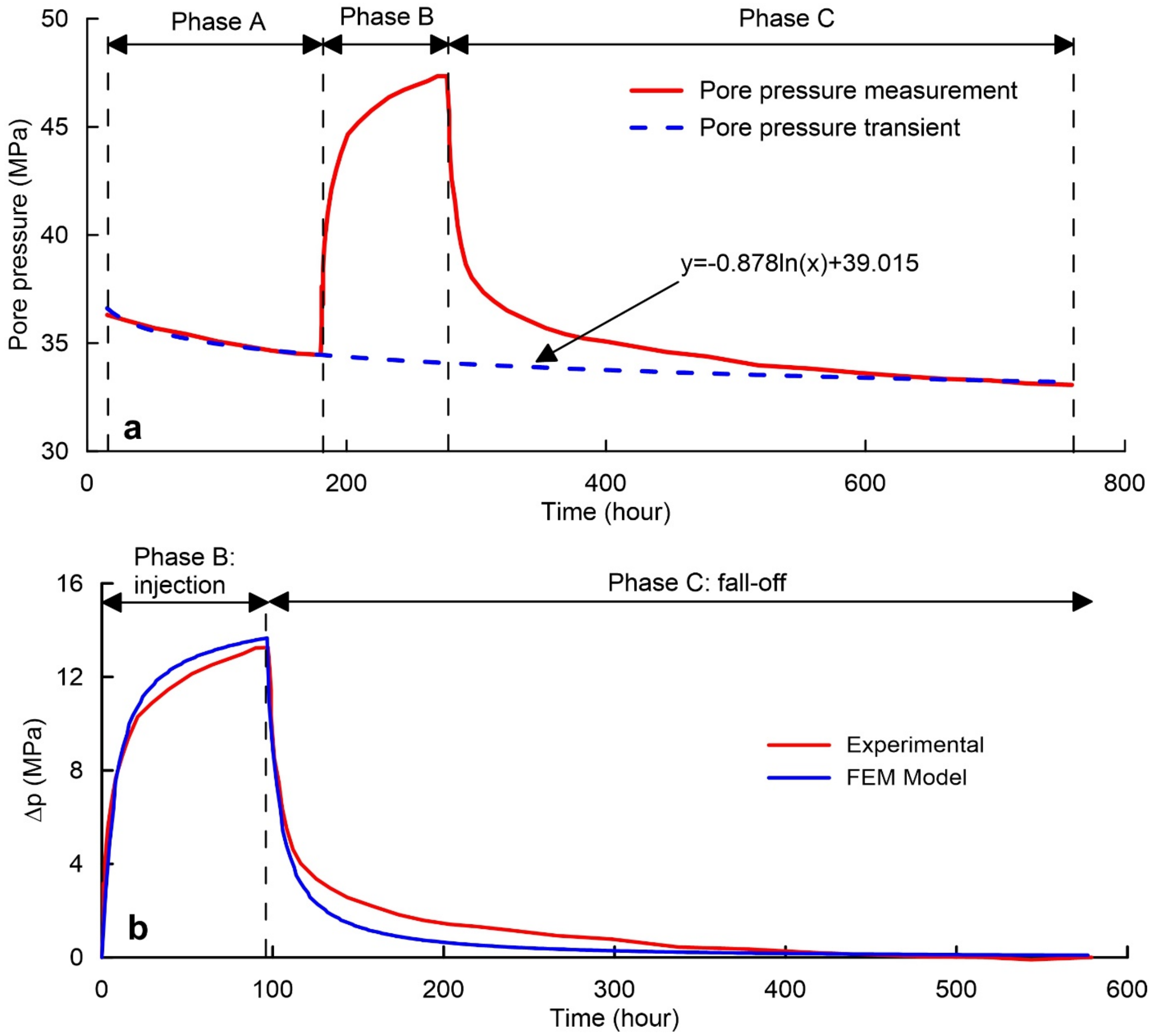


**Figure S8.** (a) Pore pressure at the memory gauge in Figure 2a during the pumping test at the Cavone 14 well[43]. The blue dotted curve represents the long wavelength change in the pore pressure from decreased injection during phase A. This long wavelength pore pressure was removed from the pore pressure measurement. (b) Comparison between the measured (red curve) and modelled (blue curve) pore pressure increases at the memory gauge in Figure 2a. This figure was created with Grapher® from Golden Software, LLC (www.goldensoftware.com).

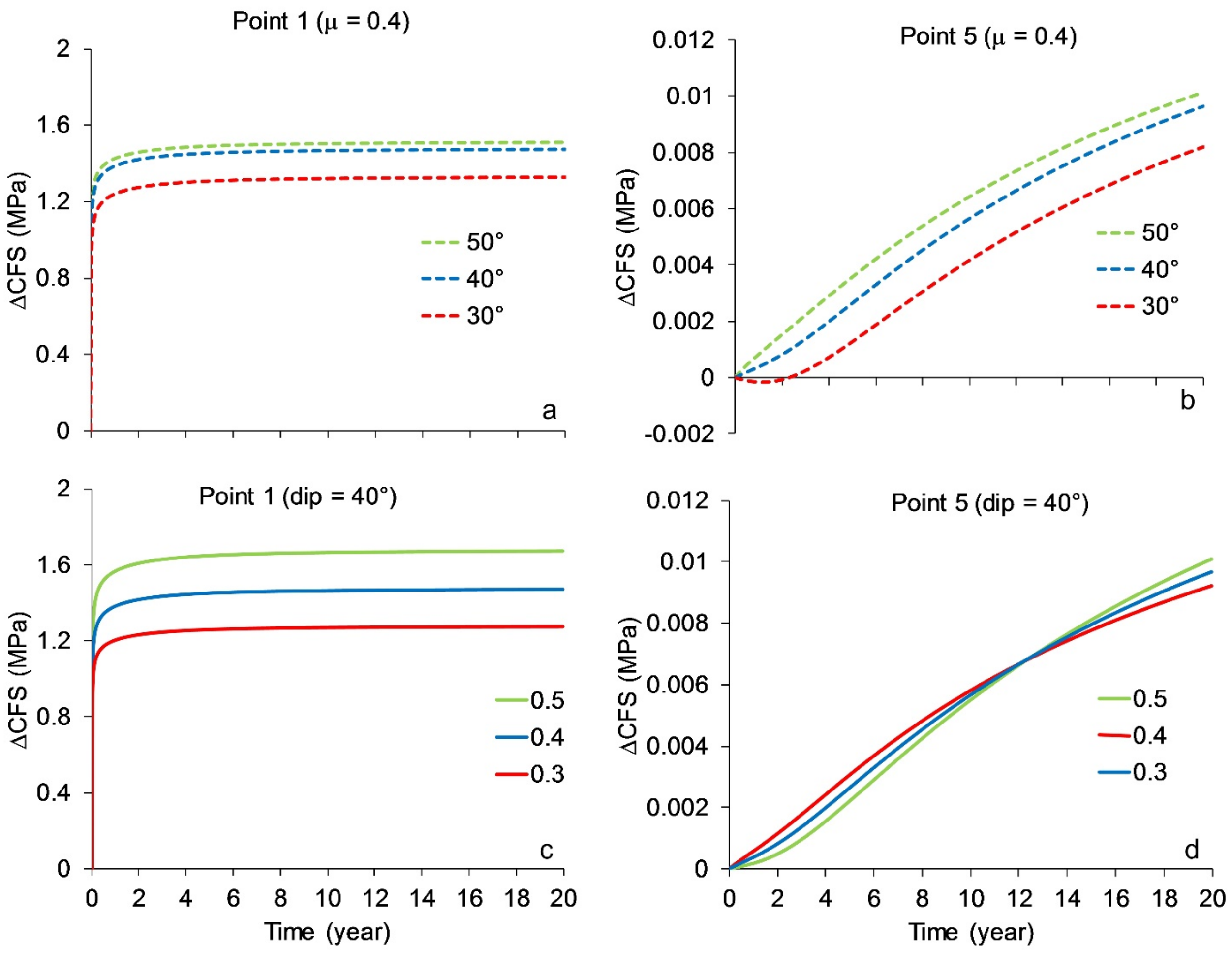


**Figure S9.** Parametric evaluation of the ΔCFS (DIFF model) at points 1 and 5 in Figure S6 with a fault dip of 30°, 40° and 50° (dashed lines) and a friction coefficient of 0.3, 0.4 and 0.5 (continuous lines). (a) ΔCFS at point 1 for a friction coefficient of 0.4 and a variable fault dip. (b) ΔCFS at point 5 for a friction coefficient of 0.4 and a variable fault dip. (c) ΔCFS at point 1 for a fault dip of 40° and a variable friction coefficient. (d) ΔCFS at point 5 for a fault dip of 40° and a variable friction coefficient. These figures were created with Grapher® from Golden Software, LLC (www.goldensoftware.com).

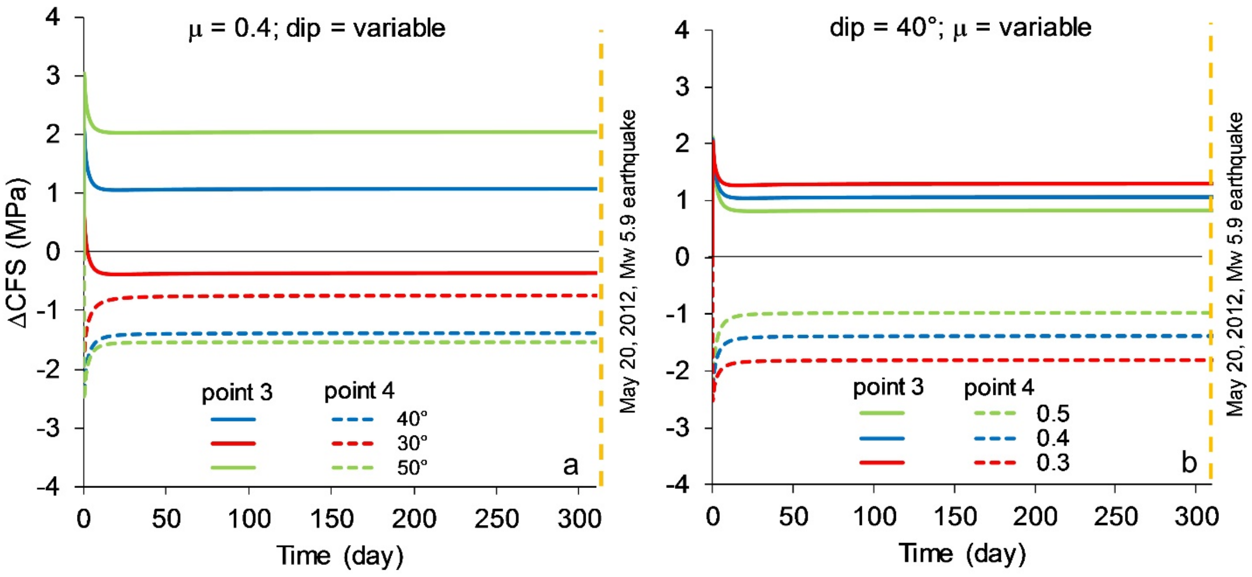


**Figure S10.** Parametric evaluation of the ΔCFS (EQK model) at points 3 and 4 in Figure 3e with a fault dip of 30°, 40° and 50° and a friction coefficient of 0.3, 0.4 and 0.5. (a) ΔCFS at points 3 and 4 for a friction coefficient of 0.4 and a variable fault dip. (b) ΔCFS at points 3 and 4 for a fault dip of 40° and a variable friction coefficient. These figures were created with Grapher® from Golden Software, LLC (www.goldensoftware.com).
